# Visualization of a Dinoflagellate-Infecting Virus HcDNAV and Its Infection Process

**DOI:** 10.3390/v10100554

**Published:** 2018-10-11

**Authors:** Yoshihito Takano, Yuji Tomaru, Keizo Nagasaki

**Affiliations:** 1 Faculty of Science and Technology, Kochi University, Nankoku 783-8502, Kochi, Japan; takadino@gmail.com; 2 National Research Institute of Fisheries and Environment of Inland Sea, Japan Fisheries Research and Education Agency, Hatsukaichi 739-0452, Hiroshima, Japan; tomaruy@affrc.go.jp

**Keywords:** viral infection, *Heterocapsa circularisquama* DNA virus (HcDNAV), dinoflagellate, virus entry, field emission scanning electron microscopy, epifluorescence microscopy, transverse groove, viroplasm, vertex protrusion, *Dinodnavirus*

## Abstract

HcDNAV (a type species of Genus *Dinodnavirus*) is a large double-stranded DNA virus, which lytically infects the bloom-forming marine microalga *Heterocapsa circularisquama* Horiguchi (Dinophyceae). In the present study, detailed observation of the HcDNAV particle and its infection process was conducted via field emission scanning electron microscopy (FE-SEM) and epifluorescence microscopy (EFM). Each five-fold vertex of the icosahedral virion was decorated with a protrusion, which may be related to the entry process of HcDNAV into the host. The transverse groove of host cells is proposed to be the main virus entry site. A visible DAPI-stained region, which is considered to be the viroplasm (virus factory), appeared in close proximity to the host nucleus at 11 h post infection (hpi); the putative viral DAPI signal was remarkably enlarged at 11–30 hpi. It was kidney-shaped at 13–15 hpi, horseshoe-shaped at 20 hpi, doughnut-shaped at 30 hpi, and changed into a three-dimensionally complicated shape at 51–53 hpi, by which time most parts of the host cell were occupied by the putative viral DAPI signal. While the virions were within the viroplasm, they were easily distinguishable by their vertex protrusions by FE-SEM.

## 1. Introduction

Dinoflagellates (Dinophyceae) are highly abundant and diverse unicellular eukaryotic microorganisms found in aquatic environments. They constitute a major class of eukaryotes within Alveolata, a firmly established deep phylogenetic lineage that includes diverse classes of protists, such as apicomplexans and ciliates [1]. About half of dinoflagellates are autotrophic using photosynthesis and half are heterotrophic using feeding with diverse mechanisms, while some are mixotrophic using both nutrition strategies. Several dinoflagellate species produce toxins that kill fish and bivalves, and contaminate edible shellfish, thereby causing serious economic damage to aquaculture related industries [2,3]. *Heterocapsa circularisquama* Horiguchi, a bloom-forming dinoflagellate, which causes high mortality rates among shellfish such as pearl oysters and mussels, is one of the most intensively studied dinoflagellate species [4,5]. At present, only two viruses infecting *H. circularisquama* have been deeply studied; one is a small single-stranded RNA virus, HcRNAV [6]; the other is a giant double-stranded DNA virus (“girus”) [7,8], HcDNAV [9,10]. Currently, HcDNAV is the sole intensively studied DNA virus isolated from the superphylum, Alveolata [11]. HcDNAV is reported to play a significant role in the disintegration process of *H. circularisquama* blooms [10,12]. HcDNAV has a large icosahedral capsid (180–210 nm in diameter) and its genome size is estimated to be ca. 356 kbp in length [9,13]. During its multiplication process, virions emerge from a specific cytoplasm compartment, called “viroplasm,” or “virus factory,” which is created by the virus. However, its infection tactics have been revealed only partially by previous studies [9,10]. To understand the host-virus relationship, a more intensive analysis of its infection process is essential. Based on such a background, field emission scanning electron microscopy (FE-SEM) and epifluorescence microscopy (EFM) were used for a detailed observation of the infection process of HcDNAV over time.

## 2. Materials and Methods

The host-virus system used in the present study comprised *H. circularisquama* HU9433-P and HcDNAV01 (previously designated as “HcV03”), which was free from bacterial contamination [9]. The host-virus system was incubated in a sterilized IMK+s medium (natural seawater enriched with Daigo’s IMK Medium (Nihon Pharmaceutical, Tokyo, Japan) and soil extract) at 22 °C and photon flux density of ca. 150 μmol photons m^−2^ s^−1^ provided by cool-white fluorescent lamps with a 12 h light and 12 h dark cycle.

An exponentially growing *H. circularisquama* culture was inoculated with HcDNAV01 suspension at a multiplicity of infection of ca. 100 (copies/cell); the virus-copy number was estimated by a real-time PCR system designed for HcDNAV DNA polymerase gene. Consequently, the host culture was lysed as previously observed [10]. In the present study, the infection process was examined as shown below. For FE-SEM, aliquots of the culture were sampled at 5, 10, and 20 min post-inoculation (mpi) and 5 days post-inoculation (dpi). The cells were then fixed with a final concentration of 0.5% of osmium tetroxide for 15–30 min at room temperature, rinsed a few times in distilled water, and dehydrated using an ethanol series (30%, 50%, 70%, 90%, 95%, and 100%). The cells were critical point dried on a polycarbonate membrane filter of 0.22- or 3.0-μm pore size (Isopore^TM^ GTTP02500 or TSTP02500, Merck-Milipore, Burlington, MA, USA) using a JEOL JCPD-5 critical-point-dryer (JEOL, Tokyo, Japan). Dried cells were coated with platinum in a JEOL JFC-1100E ion-sputter (JEOL, Tokyo, Japan). These cells were observed under an FE-SEM (JSM-6500F, JEOL, Tokyo, Japan).

For EFM, sampling was conducted at 2, 4, 6, 8, 11, 13, 15, 20, 25, 30, 36, 40, 43, 44, 48, 51, 53, and 54 h post-infection (hpi). For DAPI staining, the cells were fixed with a final concentration of 2% of glutaraldehyde in a 1.5 mL tube, stained with a final concentration of 0.1–0.5 µg/mL of DAPI (4′,6-diamidino-2-phenylindole) on a slide glass, and observed and photographed using a BX51 microscope (Olympus Co. Ltd., Tokyo, Japan) with a DP22 digital camera (Olympus Co. Ltd., Tokyo, Japan) fitted with Nomarski interference optics (Olympus Co. Ltd., Tokyo, Japan). In this experiment, uninfected cells of *H. circularisquama* were also observed by EFM in parallel for comparison.

## 3. Results and Discussion

**Figure 1:**
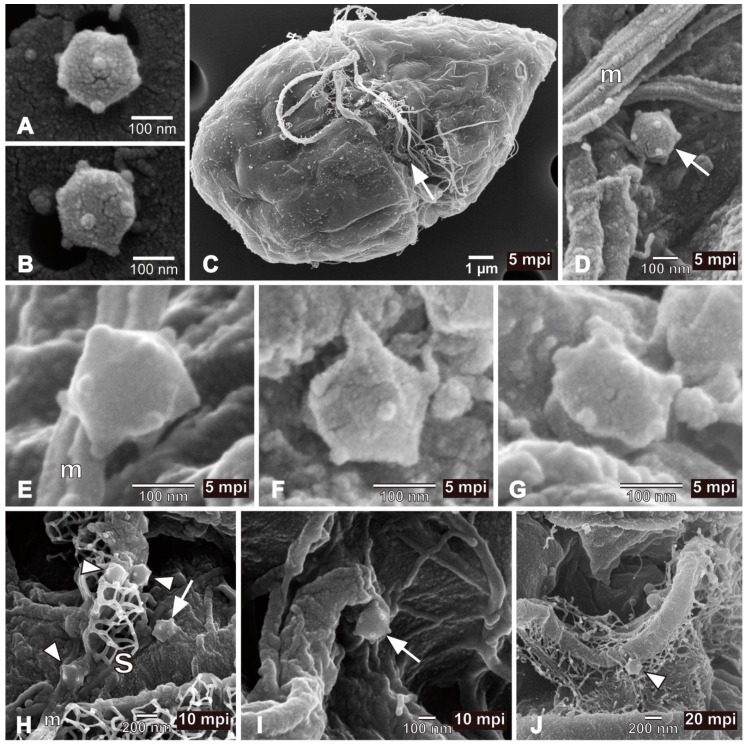

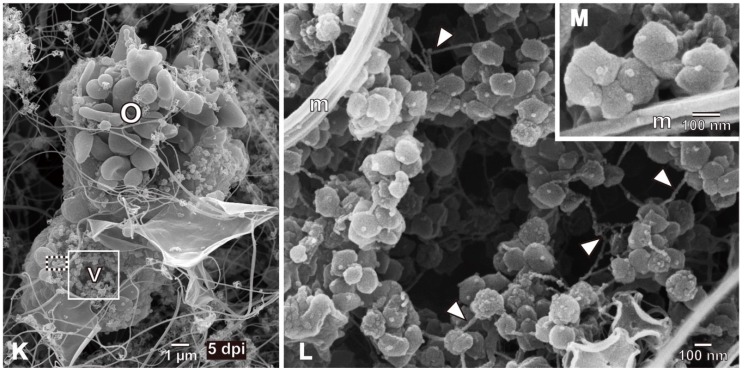
FE-SEM images of viruses and virus-infected cells. (**A**,**B**) HcDNAV particles. Because of the pentagonal (**A**) and hexagonal projection (**B**), the virion is shown to be three-dimensionally icosahedral in shape. Note that each five-fold vertex is decorated with a protrusion. (**C**) *Heterocapsa circularisquama* cell inoculated with HcDNAV at 5 mpi. An arrow indicates an HcDNAV particle attached to the host cell. (**D**) Higher magnification of the virus particle attached to the host cell shown in (**C**) (arrow). (**E**) An HcDNAV particle immediately after directly attaching to the host cell surface at 5 mpi. Note that the virion appeared to be adsorbed to the host via the protrusion. (**F**,**G**) An HcDNAV particle directly attached to the host cell surface observed from two different angles suggesting possible structural change of HcDNAV virion after adsorption to host cells at 5 mpi. (**H**) HcDNAV particles on the host cell surface; three of them with arrowheads attached to mucilage (m) excreted from the host cell and one with an arrow directly adhered to the cell surface and appears buried into the host cell at 10 mpi. (**I**) An HcDNAV particle likely being buried into the host cell at 10 mpi (arrow). (**J**) An HcDNAV particle (arrowhead) attached to the transversal flagellum of the host cell at 20 mpi; not directly adhered to the host cell surface. (**K**) A lysed entire cell of *H. circularisquama* at 5 dpi; the cell shape is apparently different from (**C**). Note that both cytoplasmic organelles (o) and viroplasm (v) are visible. (**L**) Higher magnification of the white-lined square area in (**K**), showing virus particles in the viroplasm. Note that many virus particles do not completely form icosahedral shapes (i.e., deformed shape), but are equipped with the protrusion at candidates for the vertex, and that fibrous materials (arrowheads) are also present. (**M**) Higher magnification of the black-and-white dashed lined square area in (**K**), showing close-up of immature virus particles in the viroplasm. Abbreviations, m: mucilage excreted from the host cell, o: cytoplasmic organelles, s: body scales of the host cell, characterizing *H. circularisquama*, v: viroplasm.

The resulting FE-SEM clearly showed that: the HcDNAV particle was icosahedral in shape as expected by TEM observation [9,10]; each five-fold vertex of the capsid was decorated with a protrusion structure (Figure 1A,B); viral adsorption to host cell surface occurred within at least 5 mpi (Figure 1C,D). Although the role of the vertex protrusion was not elucidated, its possible relationship to the process of virus entry into host cells was suggested (Figure 1E). Other viruses, such as bacteriophage phi174, also have vertex protrusions on the virion surface, and their function in its infection process has been intensively studied by 3D cryo-electron microscopy [14]. Endocytosis is one of the potential mechanisms following viral adsorption that may aid the entry of HcDNAV into host cells; however, there have been no reports on the endocytotic activity of *H. circularisquama*. Besides, structural change of virions into a stem-like shape following cell adsorption suggests the involvement of an entry mechanism on the virus side (Figure 1F,G). The polarity of the virion cannot be verified based only on the observation of free virus particles (Figure 1A,B). Infection by the *Chlorella*-infecting virus PBCV-1 is reported to be initiated by specific attachment with a spike oriented toward the cell wall at the unique vertex [15]. To verify whether a similar process applies to infection by HcDNAV or to determine which vertex of the virion is utilized for attachment to the host cell surface, further analysis via cryo-electron microscopy would be necessary.

At 5 and 10 mpi, most virus particles directly attached to the plasma membrane of the host cell (ca. 74%, *n* = 23) were specifically located along the transverse groove (Figure 1H [arrow] and I). The others were found not only on the peripheral area of the transverse groove, but also on the epitheca and the hypotheca (parts above and below the transverse groove, respectively). Hence, the transverse groove was proposed to be the main virus entry site; this is the first report describing the cell surface site-specific function of dinoflagellates from the viewpoint of viral entry. It was assumed that free virus particles were engulfed by the water current caused by the transverse flagellum and were physically trapped in the hollow transverse groove. Some virus particles attached to the mucilage excreted from the host cell (Figure 1H, arrowheads), or to the transverse flagellum (Figure 1J, arrowhead). As long as we observed the virus-infected cells (23 cells in total) by FE-SEM, only a sole virus particle was found to be directly attached to each host cell. Further study would be needed to discuss this interesting result.

**Figure 2:**
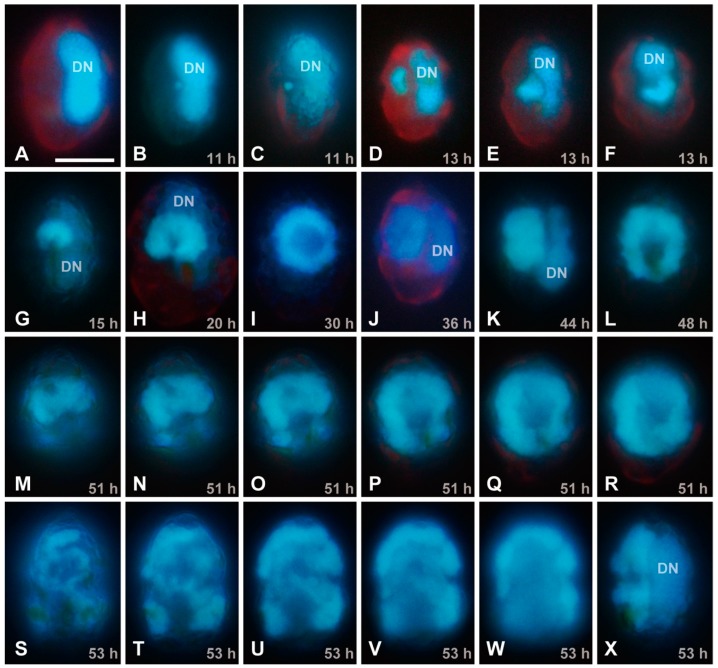
EFM images of DAPI-stained *Heterocapsa circularisquama* cells at the same magnification for observation of a putative viral region; the scale bar in (**A**) indicates 10 µm. The DN indicates a host nucleus (dinokaryon = dinoflagellate nucleus). (**A**) Lateral view of an uninfected cell, showing the elongated nucleus located at the dorsal region of the cell. (**B**–**X**) HcDNAV-infected cells at 11–53 hpi. (**B**,**C**) Lateral views of infected cells at 11 hpi. Note the small DAPI-stainable region located close by the ventral side of the middle part of the host nucleus; it is considered to be a viroplasm (**B**), which moved to the ventral region of the host cytoplasm (**C**). (**D**–**F**) Infected cells at 13 hpi. Lateral view (**D**) and lateral and apical-ventral views of the same cell (**E**,**F**). Each viroplasm becomes enlarged and kidney-shaped. (**G**) Left-ventral view of an infected cell at 15 hpi. A kidney-shaped viroplasm is enlarged. (**H**) Ventral view of an infected cell at 20 hpi. A viroplasm becomes enlarged and horseshoe-shaped. (**I**) Ventral view of an infected cell at 30 hpi. A viroplasm becomes enlarged and doughnut-shaped. (**J**) Lateral view of an infected cell at 36 hpi. A doughnut-shaped viroplasm becomes thicker. (**K**) Lateral view of an infected cell at 44 hpi. A viroplasm becomes thicker. (**L**) Ventral view of an infected cell at 48 hpi. A doughnut-shaped viroplasm becomes enlarged and oblong. (**M**–**R**) Different focal planes from surface to middle of ventral views of the same infected cell at 51 hpi. A viroplasm becomes further enlarged. (**S**–**X**) Different focal planes from surface to middle of ventral views (**S**–**W**) and lateral view (**X**) of the same infected cell at 53 hpi. Enlargement and duplexing of the viroplasm was also observed (**S**–**X**).

By means of EFM, no viral signal was detected at 0–8 hpi (Figure 2A), whereas a small DAPI-stainable region was detected next to the host nucleus at 11 hpi (Figure 2B,C). Considering that no DAPI-stainable signal was detected in uninfected cells except for the nucleus and the host does not contain any intracellular symbiotic bacteria, it is most probable that the DAPI-stainable signal is a viroplasm. The host nucleus was distinguishable from the putative viroplasm, because their chromosomes are permanently condensed throughout the cell cycle in dinoflagellate cells. The putative viral DAPI signal showed significant enlargement at 11–20 hpi (Figure 2B–H), changed into doughnut-like shape at 30–48 hpi (Figure 2I–L), and occupied the most parts of the host cells at 51–53 hpi (Figure 2M–X). Although the decaying process of the host nucleus was not clearly examined in the present study, this observation corresponds well with the TEM analysis of HcDNAV-infected cells [9,10].

**Figure 3:**
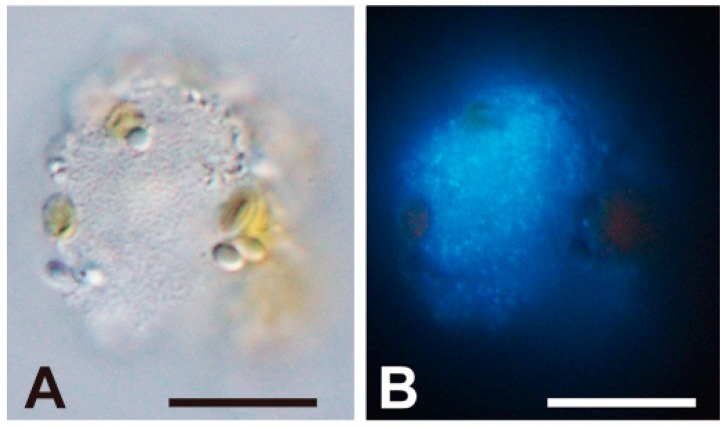
Light (**A**) and epifluorescence (**B**) microscopic images of an HcDNAV-infected *Heterocapsa circularisquama* cell at 51 hpi stained with DAPI. The scale bars indicate 10 µm. In the cell, an assembly of virus capsids presumably filled with viral DNA are distinguishable (**B**). Note its possible similarity in phase to the virally lysed cell shown in Figure 1K–M.

In the present study, FE-SEM photographs of the viroplasm were taken as well. In a host cell at 5 dpi, where progeny virus particles remained unreleased, having combined with some fibrous materials (Figure 1L, arrowheads). To our knowledge, this is the first report of a 3D-based observation of intracellular “giruses” in algal viroplasms. Although the progeny virus particles in the viroplasm were equipped with vertex protrusions, their shapes did not display the icosahedral symmetry (Figure 1L,M) suggesting that maturation of the intracellular virions may have not been completed. Some fibrous materials were also observed in the viroplasm (Figure 1L), but their composition and function are unknown. Lysed cells still harboring viroplasms were observed by EFM, in which lysis may have occasionally occurred before the maturation of intracellular progeny virus particles (Figure 3).

## 4. Conclusions

In conclusion, the infection process of HcDNAV was partially elucidated visually. To our knowledge, this is the first report describing the replication process of Alveolata-infecting viruses. Considering that its genome analysis is presently under way, the next objective is comparison between gene expression and intracellular morphological change over time, in order to further clarify infection mechanisms of dinoflagellate-infecting DNA viruses.
